# Potential Antiproliferative and Antimetastatic Effects of *Artemisia eriantha*: An In Vitro Study Focused on Hepatocarcinoma Cells

**DOI:** 10.3390/biology13120985

**Published:** 2024-11-28

**Authors:** Loretta Pace, Federica Ragusa, Lara Lizzi, Maria Giovanna Armillotta, Mara Massimi

**Affiliations:** Department of Life, Health, and Environmental Sciences, University of L’Aquila, 67100 L’Aquila, Italy

**Keywords:** HCC, *A. eriantha*, HepG2, Huh7, nutraceuticals, natural compounds

## Abstract

*Artemisia eriantha* is a subendemic species of the Central Apennine, valued locally for its applications in ancient and traditional medicine for its antibacterial, antifungal, anthelmintic, digestive, and antispasmodic effects. Several of these properties are also found in other species within the same genus, including recent findings highlighting their anti-tumor actions. However, the presence of cytotoxic or anti-tumor activity has never been studied in *A. eriantha*. The aim of this study was thus to assess the potential anti-proliferative, pro-apoptotic, and antimetastatic effects of this plant using in vitro models of hepatocarcinoma cells (HepG2 and Huh7 cells). Treatment with *A. eriantha* extract (130 μg/mL) resulted in the reduced proliferation of HepG2 and Huh7 cells, driven by the dysregulation of proteins involved in the cell cycle (such as cyclin D1, cyclin E, and p27) and the activation of the p53-p21 pathway, leading to an increase in apoptotic activity. In addition, the treatment also lowered the healing capability after scratch wounds in Huh7 cells, which are characterized by a remarkable migratory capacity, suggesting that the *A. eriantha* extract may also be effective in controlling the migration and spread of metastatic cells. In conclusion, *A. eriantha* extract 130 μg/mL appears to interfere with key factors of HepG2 and Huh7 cell proliferation and invasion and emerges as a potential new adjuvant for the prevention and/or treatment of hepatocellular carcinoma. Additional research is necessary to establish if the effects observed are cell-specific and to elucidate the mechanisms of action and signaling pathways underlying its effectiveness.

## Simple Summary

*Artemisia eriantha* (Apennine Genepì) is a subendemic species native to the Central Apennines, valued locally for its use in traditional and ancient medicine due to its antibacterial, antifungal, anthelmintic, digestive, and antispasmodic properties. Many of these benefits are shared with other species within the *Artemisia* genus, which have also recently demonstrated anti-tumor properties. However, the potential cytotoxic and/or antitumor effects of *A. eriantha* have not yet been explored. The aim of this study was to evaluate the anti-proliferative, antitumor, and anti-metastatic properties of the plant using in vitro models of liver cancer cells. Treatment with an extract of *A. eriantha* inhibited the proliferation, survival, migration, and metastatic capabilities of liver cancer cell lines. *A. eriantha* extract appears to influence key factors associated with cancer cell proliferation and invasion, positioning it as a promising candidate for further research as an adjuvant in the prevention or treatment of liver cancer.

## 1. Introduction

Hepatocellular carcinoma (HCC) is the most frequent primary malignant disease of the liver and represents the second leading world cause of cancer death, both for high incidence and unfavorable prognosis. Different molecular dysfunctions in multiple signaling pathways contribute to the development, growth, and dissemination of HCC, rendering this tumor particularly resistant to traditional systemic chemotherapy [1,2]. To reduce drug resistance phenomena, combined therapies consisting of cocktails of drugs with different mechanisms of action or targeting distinct molecular pathways have been introduced with positive results; however, mortality and recurrence remain alarmingly high. The search for new adjuvant compounds that could improve patient survival, either as standalone treatments or in combination with the currently used therapies, continues to be a priority [3,4].

Recently, research on the anticancer and/or cytotoxic effects of bioactive compounds derived from various plant species has gained a significant boost, with many efforts aimed at understanding their mechanisms of action and their pharmacological potential in the oncological field [5,6]. The genus *Artemisia* is heterogeneous, containing over 500 species [7] dispersed mainly in the temperate zones of Asia, Europe, and North America. Many species of the genus *Artemisia* have been traditionally valued and used for treating a wide range of human health conditions, including malaria, microbial infections, helminth infestations, diabetes, inflammation, and oxidative stress. These therapeutic properties are attributed to the presence of various bioactive components, such as terpenoids, coumarins, sesquiterpene lactones, lignans, alkaloids, and others [8,9,10].

Among the different species, *Artemisia annua* L. has been used for centuries in traditional Chinese medicine for its antimalarial properties, attributed to the presence of the sesquiterpene lactone artemisinin and its derivatives. Recently, *A. annua* has also gained attention for its antitumor effects [2,11,12], well established by in vivo and in vitro experiments in various cancer models, including non-small cell lung cancer [13], colorectal cancer [14], endometrial cancer [15], breast cancer [16], and hepatocellular carcinoma [17,18]. The anti-cancer effects of *A. annua* are not solely attributed to artemisinin; they result from the combined action of various bioactive compounds, including flavonoids, coumarins, phenolic acids, and terpenes, as well as sabinene, which together provide antioxidant, anti-inflammatory, and cytotoxic properties [19,20].

In recent years, significant antioxidant, anti-inflammatory, and antitumor effects have also been described for selected species within the same genus, including *A. sieversiana* [21], *A. capillaris* [22,23], *A. vulgaris* [24], *A. dracunculus* [25,26], *A. anomala* [27,28], *A. scoparia* [29], and *A. absinthium* [30,31].

In this study, our attention focused on a lesser-studied species of the genus *Artemisia*, *Artemisia eriantha* Ten. (*A. eriantha*) (synonyms: *Artemisia genipi* Weber ex Stechm. subsp. *eriantha*) (Ten.) P.Fourn.; *Artemisia petrosa* (Baumg.) Jan; *Artemisia petrosa* (Baumg.) Jan subsp. *eriantha* (Ten.) Giacom. and Pignatti; *Artemisia umbelliformis* Lam. subsp. *eriantha* (Ten.), Vallès-Xirau and Oliva Brañas [32]. This plant is a sub-endemic species of the Italian Central Apennines belonging to the Asteraceae family and is characterized by notable phytotherapeutic properties, including stimulating, digestive, and antispasmodic effects [33]. The impact of gastro-intestinal digestion on the polyphenolic compounds found in Apennines Genepì infusions, as well as their antioxidant qualities, has also been recently reported [34].

These properties are primarily due to the presence of essential oils rich in monoterpenes (e.g., α and β-thujone, α and β-pinene, p-cymene, salvene, sabinene, and camphor), sesquiterpenes (e.g., α-bisabolol, nerolidol, germacrene D, nerolidol, and β-caryophyllene), and other active ingredients, as identified through analyses conducted using high-resolution gas chromatography (HRGC) and headspace-solid phase microextraction gas chromatography mass spectrometry (HS-SPME-GC-MS). Among the monoterpenes identified in this species, salvene appears to be a distinctive component for its identification and is responsible for its characteristic scent and flavor [35]. The presence of sabinene is particularly significant, as it is found in greater concentrations compared to A. annua, where it is believed to contribute significantly to its beneficial effects [36,37].

*A. eriantha* is classified as an endangered species due to its very short reproductive period and its occurrence in a very selective environment, typically growing in rocky cracks and on limestone slopes above 2200 m, exposed to northern exposure and cold winds. Furthermore, this plant faces threats from indiscriminate harvesting by the local population and tourists, as it is considered an essential ingredient for the preparation of the namesake liqueur (Apennines’ Genepì), known for its characteristic aromatic bitterness. The EU Habitats Directive 92/43 identified *A. eriantha* as a species of European Community (EC) interest that requires strict protection.

The in vitro propagation technique thus represents an effective strategy by which to preserve this species. In fact, plants produced in vitro can be both reintroduced into their natural habitat and sold for pharmaceutical or liqueur preparations, contributing to eco-sustainable economic activities for the mountain communities. A method for the micropropagation of *A. eriantha* has been previously developed in our laboratory and subsequently improved with synthetic seeds enriched with rhizobacterial consortium [38,39]. An extended characterization and analysis of active ingredient composition and biometrical parameters confirmed that the type and levels of compounds of the in vitro regenerated plants were comparable to those obtained from wild plants; however, in vitro plantlets possessed higher content of α-bisabolol, *β*-cariophyllene, and *E*-nerolidol, with lower contents of *α*- and *β*-thujone, compared to wild or soil-grown plants. In addition, lower concentrations of α- and β-thujone were found in the leaf apices compared to the yellow flower heads of the same plant [35]. These findings are of interest as all these molecules are known for their significant antitumor activity [40,41,42], but some caution regarding dosage is recommended for thujones. Thujone concentrations in fact represent an important parameter to be considered for human health, as high doses of these terpenoid ketones are considered neurotoxic [43,44].

The aim of this research was to verify the cytotoxic, antiproliferative, and antimetastatic potential of alcoholic leaf extracts of micropropagated plants of *A. eriantha*. The study was carried out using two hepatocyte cell lines characterized by a different degree of tumor aggressiveness, namely Huh7 and HepG2 cells. Specifically, Huh7 cells represent a transformed and tumorigenic hepatocyte line, characterized by the presence of multiple mutations, including a point mutation in the p53 gene [45]; HepG2 cells, on the other hand, maintain numerous characteristics typical of normal hepatocytes and show a lower degree of tumorigenicity than Huh7 cells.

## 2. Materials and Methods

### 2.1. Collection and Preparation of A. eriantha Leaf Extracts

The leaves of *A*. *eriantha* clones were collected from the experimental field of the Alpine Botanical Garden of Campo Imperatore, University of L’Aquila (2117 m asl, Gran Sasso Monti della Laga National Park) at the end of July 2023, during the balsamic period. Immediately after collection, the leaves were separated from the stems, washed with distilled water to eliminate dust and impurities, and allowed to dry. The leaves were then broken into small pieces (0.5–1 cm), and 5 g were soaked in 100 mL of ethanol (70%) for 30 days at room temperature, protected from light. The mixture was subsequently filtered through Whatman filter paper No. 1. The volume of the filtrate was reduced to dryness at 40 °C using a rotary evaporator (Rotary Vacuum Evaporator Laborota-4010, Heidolph Co., Schwabach, Germany) to obtain crude extract, which was stored in a refrigerator, away from light, until use.

### 2.2. Cell Cultures

The HepG2 cell line was purchased from the American Type Culture Collection (ATCC, Manassas, VA, USA), while the Huh7 cell line was purchased from JCRB Cell Bank (Osaka, Japan). The proliferating cells HepG2 and Huh7 were plated on standard tissue culture-treated polystyrene plates at a density of 15 × 10^3^ and 8 × 10^3^ cells per cm^2^, respectively, and grown in Dulbecco’s Modified Eagle Medium (DMEM) (EuroClone, Milan, Italy), supplemented with 10% fetal bovine serum, 2 mM L-glutamine, 100 μg/mL streptomycin, and 100 μg/mL penicillin.

All cells were incubated in a humid atmosphere containing 5% CO_2_ at a temperature of 37 °C. The cell lines were intermittently tested for the presence of mycoplasma.

Twenty-four hours after plating, the cells were treated with *A. eriantha* ethanol extract for the time specified by the experiment. The concentration of the extract, and consequently that of the ethanol, in the culture medium was kept below 0.001%; control cells received the same amount of ethanol (vehicle).

### 2.3. Viability/Cytotoxicity Test

The viability test was performed on cells seeded in 48 multiwell (MW) plates. Twenty-four hours post plating, the cells were treated with different concentrations (50–350 µg/mL) of ethanol extracts of *A. eriantha* and analyzed after 24, 48, and 72 h. After the treatment, cell viability was assessed using the Trypan Blue dye exclusion test, as previously described [46]. The IC50 was calculated for both cell lines treated with the *Artemisia* (Immunological Sciences, Rome, Italy) *eriantha* extract for 48 h, using data from the Trypan Blue dye exclusion test and the AAT Bioquest IC50 Calculator online tool [47].

### 2.4. Western Blot Analysis

For SDS-PAGE and immunoblotting, the cells were lysed in cold 20 mM Tris-HCl buffer (pH 7.4) containing 50 mM NaCl, 10% glycerol, 5 mM EDTA, 5 mM EGTA, 1% Nonidet P-40, 2% SDS, 2 mM sodium orthovanadate, 16 μg/mL aprotinin, 10 μg/mL leupeptin, 16 μg/mL pepstatin, and 1 mM PMSF, as previously described [48]. The lysate was passed through a syringe gauge needle to break down the DNA and then centrifuged at 12,000 rcf at 4 °C for 30 min. The supernatant was subsequently collected for protein quantification and Western blot analysis. Following protein determination using the Micro-BCA assay (Pierce™ BCA Protein Assay Kit, Thermo Scientific™, Rockford, IL, USA), 20–30 μg of proteins were heated at 90–100 °C for 5 min and then electrophoresed on 10–12% polyacrylamide gel in SDS under reducing conditions. After the protein transfer, the nitrocellulose membrane was treated with a solution containing 5% fat-free milk protein in TBS (50 mM Tris HCl pH 7.5, 50 mM NaCl, 0.1% Tween20, H_2_O) to saturate non-specific binding sites. The membrane was incubated overnight at 4 °C with the primary antibody of interest: mouse monoclonal anti-β actin 1:5000 (Sigma, St. Louis, MO, USA), mouse anti-GAPDH 1:10,000 (Immunological Sciences, Rome, Italy), mouse anti-β tubulin 1:1000 (Santa Cruz Biotechnology, Dallas, TX, USA), monoclonal anti-cleaved (89KDa) PARP-1α 1:1000 (Cell Signaling, Danvers, MA, USA), rabbit monoclonal anti-cyclin D1 1:1000 (Abcam), mouse monoclonal anti-p53 1:500 (Immunological Sciences), mouse monoclonal anti-p21 waf1/cip1 1:1000 (Cell Signaling), mouse mono clonal anti-p27kip1 1:500 (Abnova, Taipei, Taiwan), rabbit polyclonal anti-BAX 1:1000 (Immunological Sciences), rabbit monoclonal β-catenin 1:2000 (St John’s Laboratory, London, UK), rabbit polyclonal anti-TWIST1 (Cell Signaling), and polyclonal anti-PCNA 1:1000 (Immunological Sciences). After thorough washing, the membranes were incubated with the secondary antibody (anti-rabbit or anti-mouse) conjugated with alkaline phosphatase (Jackson ImmunoResearch, Cambridgeshire, UK) for 1 h. Quantitative analysis of the bands was performed on digital images using specific ImageJ 1.8.0 software (NIH, Bethesda, MD, USA), and the relative densities were normalized with respect to β-actin and/or GAPDH. All uncropped Western blot figures can be found in the Supplementary Information File.

### 2.5. Wound Healing Assay

Cell migration was visualized and analyzed using IncuCyte equipment (Sartorius Brand, Göttingen, Germany). The Wound-Healing IncuCyte Live Cell Analysis System (Sartorius Brand) provides an integrated solution for the real-time evaluation of cell morphology and migration in a scratch test (scratch wound). The images provide a clear view of the initial scratch and its subsequent incremental closing over time. In addition, the IncuCyte S3 V2018C software (Sartorius Brand) allows direct measurement of the cell density in the area affected by the scratch. The data are reported in RWD (Relative Wound Density).

### 2.6. Statistical Analysis

Each set of experiments was repeated at least in triplicate, and the standard deviation was calculated. Data are shown with mean values ± SD. For statistical analysis, the Student’s Two-Tailed *t*-Test was used, and *p* < 0.05 (*), *p* < 0.01 (**), and *p* < 0.001 (***) were considered significant values.

## 3. Results

### 3.1. Artemisia eriantha Slows Down the Proliferation of HCC Cells

The HepG2 and Huh7 cell lines were treated with different concentrations (100–350 μg/mL) of *A. eriantha* leaf extract to evaluate its effects on cell proliferation and/or toxicity.

**Figure 1:**
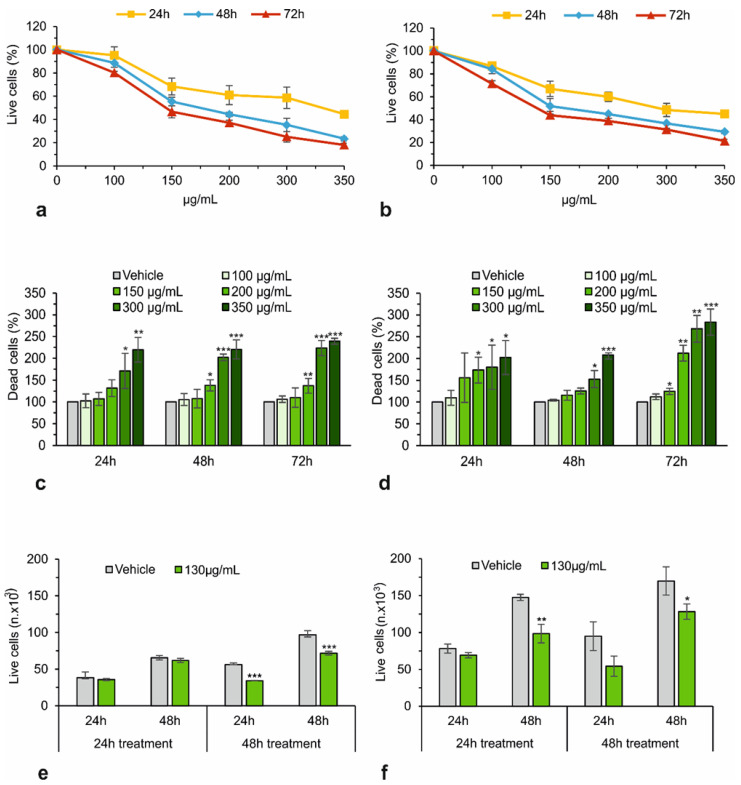
The effect of *A. eriantha* alcoholic extract on the viability of HCC cells. Dose-response effect on HepG2 (**a**,**c**) or Huh7 (**b**,**d**) cell viability (**a**,**b**) and mortality (**c**,**d**) at 24, 48, and 72 h of treatment. Reversibility test in HepG2 (**e**) and Huh7 (**f**). Cells were treated for 24 or 48 h with 130 µg/mL of extract, then washed and counted. Cells from each incubation condition were seeded again in a fresh extract-free medium and counted 24 and 48 h later. Data are the mean ± SD of at least three independent experiments. Student’s *t*-test. * *p* < 0.05; ** *p* < 0.01; *** *p* < 0.001 versus control cells (vehicle).

*A. eriantha* extract inhibits the number of viable HepG2 and Huh7 cells in a dose- and time-dependent manner, with an IC_50_ of 141 μg/mL and 127 μg/mL, respectively [Figure 1a,b]. Staining with Trypan Blue dye allowed us to calculate the number of necrotic cells and thus the potential toxicity of the extracts after 48 h of treatment. Necrotic cell death was not substantial at concentrations of up to 150 μg/mL, but it became quite significant when the concentrations were increased to 300–350 μg/mL [Figure 1c,d]. Considering the similar IC50 values for the two experimental groups (HepG2 and Huh7 cells) and the fact that using a consistent concentration can simplify the experimental design while minimizing potential discrepancies, the concentration of 130 μg/mL was chosen for all subsequent experiments with both cell lines. To assess whether the observed effects on cell growth would persist even after removal of the extract, the number of viable cells was also evaluated after cell replating in fresh culture medium without extract, as described by Pollio et al. [49]. HepG2 and Huh7 cells were incubated for 24 h and 48 h with the extracts, as described above; then, the surviving cells were washed, replenished with fresh medium, plated again, and further analyzed 24 h and 48 h later. As shown in Figure 1e, the effect of the extract is not evident in HepG2 cells treated for 24 h and observed 24 h or 48 h later, while it becomes significant 48 h before treatment. In contrast, Huh7 cells also respond to a 24-h treatment when analyzed 48 h later [Figure 1f]. This result suggests that the inhibition of proliferation is conserved in time after the removal of the extract at least up to 48 h, with HepG2 slightly more resistant than Huh7 cells, in agreement with cell growth inhibition studies. However, further studies with longer culture periods are required to verify how long the effect is maintained.

### 3.2. A. eriantha Extract Interferes with the Expression of Key Effectors of Cell Cycle Progression and Apoptosis of HCC Cells

**Figure 2:**
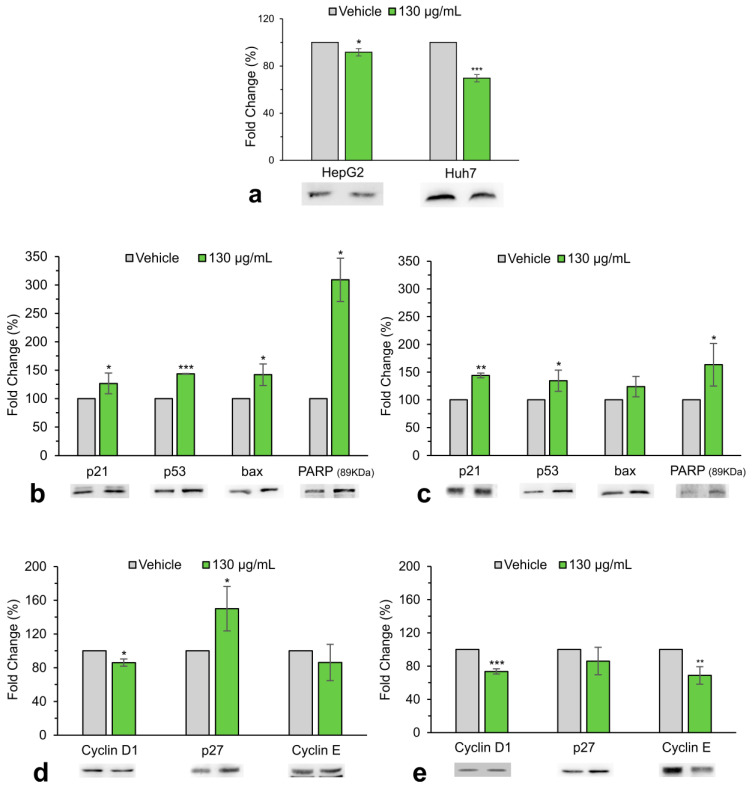
Western blot analysis of proteins involved in cell cycle progression and apoptosis and relative quantification after 48 h of treatment on HepG2 (**a**,**b**,**d**) and Huh7 cells (**a**,**c**,**e**). β-actin, β-tubulin, or GAPDH were used as internal controls. Data are the mean ± SD of three independent experiments. Student’s *t*-test. * *p* < 0.05; ** *p* < 0.01; *** *p* < 0.001 versus control cells (vehicle). (The full-length blots are included in the Supplementary Information File).

The effect of the extracts of *A. eriantha* on cell proliferation was first evaluated by studying the expression of proliferating nuclear antigen (PCNA), a nuclear protein that plays a key role in double-stranded DNA synthesis and is present at high levels in many types of cancer, including hepatocellular carcinoma. The expression of this protein was decreased after treatment, especially in high proliferative Huh7 cells, suggesting a slowdown of the cell cycle [Figure 2a]. Treatment with *A. eriantha* extract also caused a significant decrease in the protein expression of cyclin D1 and cyclin E in both cell lines, while the increase in p27 protein was only significant in HepG2 cells [Figure 2d,e].

Immunoblots also showed a significant increase in p53 and p21, proteins involved in a transitory blockage of the cell cycle and in the ultimate induction of apoptosis [Figure 2b,c]. In addition, immunoblot revealed an increase in the proapoptotic protein Bax, as well as in the cleavage of poly (adenosine diphosphate-ribose) polymerase-1 (PARP-1) in both of the cell lines analyzed [Figure 2b,c]. Members of the poly-ADP-ribose polymerases (PARP) family are involved in DNA repair, in DNA replication, and in the activation of apoptotic pathways as a cell protection mechanism against DNA damage.

### 3.3. A. eriantha Extract Reduces the Migration of HCC Cells

Cell migration is one of the hallmarks of cancer and plays a key role in the onset of metastasis. Understanding the mechanisms involved in the invasion of cancer cells could limit tumor progression and, consequently, reduce mortality for many cancer patients.

**Figure 3:**
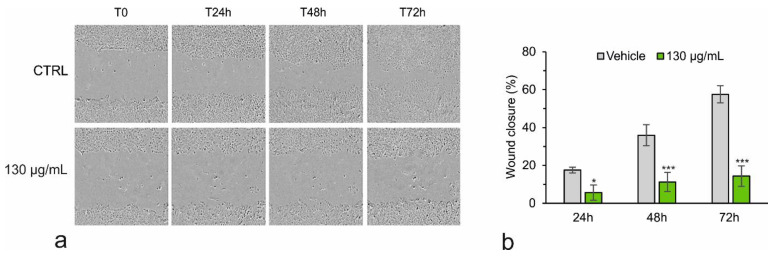
Wound healing assay for Huh7 cells using Incucyte software (Sartorius Brand). Representative images and relative wound closure values of Huh7 cells after 0, 24, 48, and 72 h of treatment (**a**,**b**). Student’s *t*-test. * *p* < 0.05; *** *p* < 0.001 versus control cells (vehicle).

To this end, the highly proliferating and migrating Huh7 cells were grown in monolayer to 80% confluence, subjected to scratch wounds, and treated with *A. eriantha* extract. After treatment, the cells were visualized at time points of 0, 24, 48, and 72 h, as well as subjected to real-time evaluation of the cell morphology during the “wound healing” test using the IncuCyte Live Cell Analysis System (Sartorius Brand). Images show that cell density in the area subjected to scratching, as measured using the Incucyte software (S3 V2018C—Sartorius Brand), is lower in the treated than in the control cells, indicating the inhibition of cell migration after treatment [Figure 3a,b].

**Figure 4:**
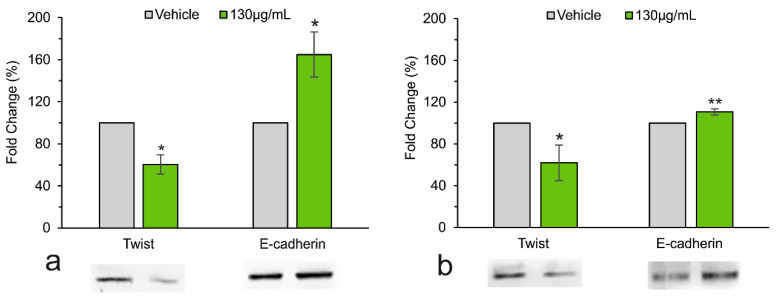
Western blot analysis and relative quantification of proteins involved in adherens junction functions and in the metastasizing process in HepG2 (**a**) and Huh7 (**b**) cells, cultured for 48 h in the absence (Vehicle) or in the presence of extract. GAPDH was used as an internal control. Data are the mean ± SD of three independent experiments. Student’s *t*-test. * *p* < 0.05; ** *p* < 0.01 versus control cells (Vehicle). (The full-length blots are included in the Supplementary Information File).

Western blot experiments [Figure 4a,b], run in parallel with the same pool of cells, showed an increase in E-cadherin, an essential component of adherens junctions with key functions in the stability of the differentiated epithelial tissues, and a decrease in Twist, a protein directly implicated in the epithelial-mesenchymal transition process and in cell metastatic processes. *A. eriantha* extract used at 130 μg/mL has a significant property in terms of stemming migration and possible metastasis of HCC cells. Additional experiments are necessary to identify the main signaling pathway effectors specifically influenced by *A. eriantha* components.

## 4. Discussion

Several studies have shown that extracts of many species of the genus *Artemisia*, one of the largest genera in the Asteraceae family, possess antitumor activity due to the presence of many different bioactive components, such as monoterpenes, sesquiterpenes, and phenolic compounds, which act via various mechanisms of action.

Previous studies by Pace et al. [35] showed that many of these components are present in *Artemisia eriantha* and maintained in micropropagated plants. Most of these constituents are monoterpenes, which include sabinene and thujones, and sesquiterpenes, which include *β*-cariophyllene and nerolidol [35]. All these components have been brought to the attention of the scientific literature as potential antitumor drugs.

The main objective of this research was thus to test the antitumor effects of leaf extract of *Artemisia eriantha* in two different HCC cell lines, HepG2 and Huh7 cell lines, and to evaluate their potential applicability as adjuvant in current HCC therapies.

In the present study, the treatment of HepG2 and Huh7 cell lines with ethanolic extract of leaf *A. eriantha*, used at a concentration of 130 µM, resulted in reduced HCC cell growth by anti-proliferative and pro-apoptotic effects, with a low degree of cell damage, as evidenced by the relatively low percentage of necrotic cells. The anti-tumorigenic in vitro effects are associated with the modulation of key effectors capable of interfering not only with the progression of the cell cycle but also with the apoptotic process and migration of the human HCC cells, HepG2 and Huh7.

The extract halved the cell population by decreasing the expression of cyclins D1 and E while inducing the expression of p27, which is essential for slowing the transition of cells from the G1 phase to the S phase. Additionally, the extract influenced the p21-p53 axis, correlating with a transient blockade of the cell cycle and the induction of apoptosis.

As is known, p53 has the function of regulating and/or inducing the transcription of different genes, including the p21 gene, a cyclin-dependent kinase inhibitor, and the main target of p53. By binding to the G1/S-Cdk and S-Cdk complexes, this inhibits their activity, thus supporting the blockage of the cell cycle and allowing the cell to repair damage at the DNA level [50]. If the damage is irreversible, the p53 protein induces apoptosis by triggering the transcription of specific target genes. Mutations of this protein are found in at least 50% of human tumors, including hepatocellular carcinoma [51]. The activation of the apoptotic process was confirmed by the induced expression of the proapoptotic protein Bax and the cleaved poly (adenosine diphosphate-ribose) polymerase-1 (PARP-1). While Bax expression correlates well with mitochondrial caspase-dependent apoptosis, the increase in cleaved PARP-1 indicates DNA fragmentation that may be triggered by both a caspase-dependent and a caspase-independent apoptotic program [52]. In addition, cleavage of PARP-1 can also be considered a downstream effect of the p53 upregulation, as shown in p53 wild-type HCT116 human colorectal cancer cells treated with an *Artemisia annua* methanol extract [53]. In our systems, this observation could explain the strongest induction of the cleaved PARP-1 in p53-wild type HepG2 cells when compared to p53-mutated Huh7 cells.

Treatment with *A. eriantha* extract also significantly reduced the migratory capacity of Huh7 cells by inhibiting the epithelial-mesenchymal transition process. This process starts from a stimulus that activates a series of signaling pathways leading to cellular polarization, loss of cell-to-cell and cell-to-matrix contacts, and rapid reorganization of the cytoskeleton. Twist exerts its effects, at least in part, by inhibiting the expression of cadherins, in particular E-cadherin. Therefore, the reduction in Twist and the increase in E-cadherin protein expression are correlated with decreased cell malignancy.

All these effects could be interpreted as the result of a synergy between the different components, of which an apoptotic, antitumor, and/or antimetastatic action has already been described.

As stated above, the sesquiterpenes nerolidol, β-cariophyllene, and α-bisabolol are major constituents of *A. eriantha*. Research carried out on human laryngeal carcinoma Hep-2 cells has demonstrated that nerolidol, also known as peruviol, acts synergistically with cisplatin in the induction of early apoptosis by activating the mitochondria-mediated apoptotic pathway and acting on the production of ROS [42]. Similar effects have been reported for β-cariophyllene in lung cancer cell lines [54].

As for α-bisabolol, this compound was also found to be cytotoxic in several human tumor cell lines, including PC-3, Hela, ECA-109, and HepG2 cells. In HepG2 cells, it induces apoptosis in a dose- and time-dependent manner, acting through both the intrinsic and extrinsic pathways, involving p53 and NFkB [40]. Some data in the literature also suggest a possible effect of α-bisabolol on the EMT mechanism, malignancy, and invasiveness, although these aspects require further investigation [55].

Data on antiproliferative and antitumor effects are also abundant for monoterpenes. Sabinene, commonly used in the perfume and flavor industries, has shown effectiveness against non-small cell lung cancer [56]. The antiproliferative effects of p-cymene, found in numerous cancer cell models, appear to be associated with the induction of the autophagic process, as well as apoptosis, to reduce the survival of tumor cells. The effect on apoptosis has been shown to be mediated by the inhibition of telomerase activity and mitochondrial dysfunction [57]. Also notable is the observation that even at extremely high concentrations, cymene shows low cytotoxicity and lethality, characteristics that make it a potential candidate as an antitumor drug [58]. Finally, Thujone, the main compound of this plant species, has also a strong, well-documented anti-tumor potential achieved by inhibiting cell viability and invasiveness while triggering apoptosis, with mechanisms overlapping those described for the other constituents of *A. eriantha* [59].

In summary, the extract of *Artemisia eriantha* contains a variety of constituents, many of which are also found in other species of the same genus, although in different proportions. These substances may play a role in modulating the cell cycle, apoptosis, and the migration of hepatic tumor cells. It is important to note, however, that the effects of an extract cannot be easily attributed to single components, as they result from the overall interaction of the multiple substances it contains, including those that may appear pharmacologically inactive. In fact, even the constituents considered inert when analyzed separately can influence the absorption, stability, and release of specific active principles within the phytocomplex, thereby impacting its availability, toxicity, and biological activity. For these reasons, the concept of synergy in complex natural mixtures and in the design of new pharmacological adjuvants is deservedly gaining increasing attention and relevance in current scientific research [60,61].

## 5. Conclusions

The present study highlighted, for the first time, that *A. eriantha* extract can interfere at various levels with key factors in the proliferation and invasion of cancerous hepatocytes and therefore represents a very important first step in the understanding of the potential of this plant species. This suggests that this plant extract could be considered among the alternative adjuvant strategies that could contribute to improving the effectiveness of current HCC therapies. Although the effectiveness in reducing cell proliferation and invasiveness has been previously reported for other plants of the genus *Artemisia*, our findings represent the first evidence for the efficacy of *Artemisia eriantha*. Studies are currently underway to better understand the potential of *A. eriantha*’s impact on human health, as well as to further analyze the molecular mechanisms that determine its efficacy and could support its use as an adjuvant in the prevention and treatment of human disorders and diseases, including HCC.

## Data Availability

The datasets used and/or analysed during the current study are available from the corresponding author upon reasonable request.

## Supplementary Materials

The following supporting information can be downloaded at: https://www.mdpi.com/article/10.3390/biology13120985/s1.

## References

[1] Llovet J.M., Zucman-Rossi J., Pikarsky E., Sangro B., Schwartz M., Sherman M., Gores G. (2016). Hepatocellular Carcinoma. Nat. Rev. Dis. Primers.

[2] Chen Z., Xie H., Hu M., Huang T., Hu Y., Sang N., Zhao Y. (2020). Recent Progress in Treatment of Hepatocellular Carcinoma. Am. J. Cancer Res..

[3] Ragusa F., Panera N., Cardarelli S., Scarsella M., Bianchi M., Biagioni S., Giorgi M., Alisi A., Massimi M. (2021). Phosphodiesterase 4d Depletion/Inhibition Exerts Anti-Oncogenic Properties in Hepatocellular Carcinoma. Cancers.

[4] Akateh C., Black S.M., Conteh L., Miller E.D., Noonan A., Elliott E., Pawlik T.M., Tsung A., Cloyd J.M. (2019). Neoadjuvant and Adjuvant Treatment Strategies for Hepatocellular Carcinoma. World J. Gastroenterol..

[5] Massimi M., Tomassini A., Sciubba F., Sobolev A.P., Devirgiliis L.C., Miccheli A. (2012). Effects of Resveratrol on HepG2 Cells as Revealed by 1H-NMR Based Metabolic Profiling. Biochim. Biophys. Acta Gen. Subj..

[6] Ai Y., Zhao Z., Wang H., Zhang X., Qin W., Guo Y., Zhao M., Tang J., Ma X., Zeng J. (2022). Pull the Plug: Anti-Angiogenesis Potential of Natural Products in Gastrointestinal Cancer Therapy. Phytother. Res..

[7] Watson L.E., Bates P.L., Evans T.M., Unwin M.M., Estes J.R. (2002). Molecular Phylogeny of Subtribe Artemisiinae (Asteraceae), Including *Artemisia* and Its Allied and Segregate Genera. BMC Evol. Biol..

[8] Bora K.S., Sharma A. (2011). The Genus *Artemisia*: A Comprehensive Review. Pharm. Biol..

[9] Bisht D., Kumar D., Kumar D., Dua K., Chellappan D.K. (2021). Phytochemistry and Pharmacological Activity of the Genus *Artemisia*. Arch. Pharm. Res..

[10] Hussain M., Thakur R.K., Khazir J., Ahmed S., Khan M.I., Rahi P., Peer L.A., Vppalayam Shanmugam P., Kaur S., Raina S.N. (2023). Traditional Uses, Phytochemistry, Pharmacology, and Toxicology of the Genus *Artemisia* L. (Asteraceae): A High-Value Medicinal Plant. Curr. Top. Med. Chem..

[11] Lang S.J., Schmiech M., Hafner S., Paetz C., Steinborn C., Huber R., El Gaafary M., Werner K., Schmidt C.Q., Syrovets T. (2019). Antitumor Activity of an *Artemisia annua* Herbal Preparation and Identification of Active Ingredients. Phytomedicine.

[12] Efferth T. (2017). From Ancient Herb to Modern Drug: *Artemisia annua* and Artemisinin for Cancer Therapy. Semin. Cancer Biol..

[13] Fu C., Zhang K., Wang M., Qiu F. (2022). Casticin and Chrysosplenol D from *Artemisia annua* L. Induce Apoptosis by Inhibiting Topoisomerase IIα in Human Non-Small-Cell Lung Cancer Cells. Phytomedicine.

[14] Jung E.J., Kim H.J., Shin S.C., Kim G.S., Jung J.M., Hong S.C., Kim C.W., Lee W.S. (2023). *Artemisia annua* L. Polyphenols Enhance the Anticancer Effect of β-Lapachone in Oxaliplatin-Resistant HCT116 Colorectal Cancer Cells. Int. J. Mol. Sci..

[15] Guo W., Wang W., Lei F., Zheng R., Zhao X., Gu Y., Yang M., Tong Y., Wang Y. (2023). Identifying the Main Components and Mechanisms of Action of *Artemisia annua* L. in the Treatment of Endometrial Cancer Using Network Pharmacology. ACS Omega.

[16] Lang S.J., Schmiech M., Hafner S., Paetz C., Werner K., El Gaafary M., Schmidt C.Q., Syrovets T., Simmet T. (2020). Chrysosplenol d, a Flavonol from *Artemisia annua*, Induces Erk1/2-Mediated Apoptosis in Triple Negative Human Breast Cancer Cells. Int. J. Mol. Sci..

[17] Zhang C.Z., Zhang H., Yun J., Chen G.G., Lai P.B.S. (2012). Dihydroartemisinin Exhibits Antitumor Activity toward Hepatocellular Carcinoma in Vitro and in Vivo. Biochem. Pharmacol..

[18] Zhang S., Mo Z., Zhang S., Li X. (2021). A Network Pharmacology Approach to Reveal the Underlying Mechanisms of *Artemisia annua* on the Treatment of Hepatocellular Carcinoma. Evid.-Based Complement. Altern. Med..

[19] Ferreira J.F.S., Luthria D.L., Sasaki T., Heyerick A. (2010). Flavonoids from *Artemisia annua* L. As Antioxidants and Their Potential Synergism with Artemisinin against Malaria and Cancer. Molecules.

[20] Kolesar J.M., Seeberger P.H. (2022). Editorial: Anticancer Potential of *Artemisia annua*. Front. Oncol..

[21] Nuermaimaiti M., Turak A., Yang Q., Tang B., Zang Y., Li J., Aisa H.A. (2021). Sesquiterpenes from *Artemisia sieversiana* and Their Anti-Inflammatory Activities. Fitoterapia.

[22] Kim J., Jung K.H., Yan H.H., Cheon M.J., Kang S., Jin X., Park S., Oh M.S., Hong S.S. (2018). *Artemisia capillaris* Leaves Inhibit Cell Proliferation and Induce Apoptosis in Hepatocellular Carcinoma. BMC Complement. Altern. Med..

[23] Kwon H., Jung J.W., Lee Y.C., Ryu J.H., Kim D.H. (2018). Chinese Journal of Natural Medicines Neuroprotective Effect of the Ethanol Extract of *Artemisia capillaris* on Transient Forebrain Ischemia in Mice via Nicotinic Cholinergic Receptor. Chin. J. Nat. Med..

[24] Zamarioli L.D.S., Santos M.R.M., Erustes A.G., Meccatti V.M., Pereira T.C., Smaili S.S., Marcucci M.C., Oliveira C.R., Pereira G.J.S., Bincoletto C. (2024). *Artemisia vulgaris* Induces Tumor-Selective Ferroptosis and Necroptosis via Lysosomal Ca^2+^ Signaling. Chin. J. Integr. Med..

[25] Sahakyan N., Andreoletti P., Cherkaoui-Malki M., Petrosyan M., Trchounian A. (2021). *Artemisia dracunculus* L. Essential Oil Phytochemical Components Trigger the Activity of Cellular Antioxidant Enzymes. J. Food Biochem..

[26] Țicolea M., Pop R.M., Pârvu M., Usatiuc L.O., Uifălean A., Ranga F., Pârvu A.E. (2024). Phytochemical Composition Antioxidant and Anti-Inflammatory Activity of *Artemisia dracunculus* and *Artemisia abrotanum*. Antioxidants.

[27] Hong F., Zhao M., Xue L.L., Ma X., Liu L., Cai X.Y., Zhang R.J., Li N., Wang L., Ni H.F. (2022). The Ethanolic Extract of *Artemisia anomala* Exerts Anti-Inflammatory Effects via Inhibition of NLRP3 Inflammasome. Phytomedicine.

[28] Albaqami J.J., Benny T.P., Hamdi H., Altemimi A.B., Kuttithodi A.M., Job J.T., Sasidharan A., Narayanankutty A. (2022). Phytochemical Composition and In Vitro Antioxidant, Anti-Inflammatory, Anticancer, and Enzyme-Inhibitory Activities of *Artemisia nilagirica* (C.B. Clarke) Pamp. Molecules.

[29] Boudreau A., Richard A.J., Harvey I., Stephens J.M. (2022). *Artemisia ccoparia* and Metabolic Health: Untapped Potential of an Ancient Remedy for Modern Use. Front. Endocrinol..

[30] Sohail J., Zubair M., Hussain K., Faisal M., Ismail M., Haider I., Mumtaz R., Khan A.A., Khan M.A. (2023). Pharmacological Activities of *Artemisia absinthium* and Control of Hepatic Cancer by Expression Regulation of TGFβ1 and MYC Genes. PLoS ONE.

[31] He M., Yasin K., Yu S., Li J., Xia L. (2023). Total Flavonoids in *Artemisia absinthium* L. and Evaluation of Its Anticancer Activity. Int. J. Mol. Sci..

[32] Acta Plantarum Artemisia Eriantha Ten Scheda IPFI. https://www.actaplantarum.org/flora/flora_info.php?id=953.

[33] Vouillamoz J.F., Carlen C., Taglialatela-Scafati O., Pollastro F., Appendino G. (2015). The Génépi *Artemisia* Species. Ethnopharmacology, Cultivation, Phytochemistry, and Bioactivity. Fitoterapia.

[34] Rocchi R., Pellegrini M., Pittia P., Pace L. (2024). Wild and Micropropagated *Artemisia eriantha* Infusions: In Vitro Digestion Effects on Phenolic Pattern and Antioxidant Activity. Plants.

[35] Pace L., Grandi S., Marotti M., Piccaglia R., Pacioni G., Spanò L. (2010). Terpenoid Profiles of in vitro Regenerated *Artemisia petrosa subsp. eriantha* (Apennines’ Genepì). Ann. Appl. Biol..

[36] Rana V.S., Amparo Blázquez M., Maiti S. (2013). Essential Oil Composition of *Artemisia annua* L. at Different Growth Stages. J. Spices Aromat. Crops.

[37] Fasciani P., Marcozzi G., Reale S., Pace L. (2017). Volatile Compounds of Ex Vitro and Wild Plantlets of *Artemisia umbelliformis* subsp. *eriantha* (Apennines’ Genepì). Acta Hortic..

[38] Pace L., Pacioni G., Spano L. (2004). In Vitro Propagation of *Artemisia petrosa* ssp. eriantha: Potential for the Preservation of an Endangered Species. Plant Biosyst..

[39] Pellegrini M., Ercole C., Di Zio C., Matteucci F., Pace L., Del Gallo M. (2020). In Vitro and in Planta Antagonistic Effects of Plant Growth-Promoting Rhizobacteria Consortium against Soilborne Plant Pathogens of *Solanum tuberosum* and *Solanum lycopersicum*. FEMS Microbiol. Lett..

[40] Chen W., Hou J., Yin Y., Jang J., Zheng Z., Fan H., Zou G. (2010). α-Bisabolol Induces Dose- and Time-Dependent Apoptosis in HepG2 Cells via a Fas- and Mitochondrial-Related Pathway, Involves P53 and NFκB. Biochem. Pharmacol..

[41] Fidyt K., Fiedorowicz A., Strządała L., Szumny A. (2016). β-Caryophyllene and β-Caryophyllene Oxide—Natural Compounds of Anticancer and Analgesic Properties. Cancer Med..

[42] Balakrishnan V., Ganapathy S., Veerasamy V., Duraisamy R., Jawaharlal S., Lakshmanan V. (2022). Nerolidol Assists Cisplatin to Induce Early Apoptosis in Human Laryngeal Carcinoma Hep 2 Cells through ROS and Mitochondrial-Mediated Pathway: An in Vitro and in Silico View. Food Biochem..

[43] Walch S.G., Kuballa T., Stühlinger W., Lachenmeier D.W. (2011). Determination of the Biologically Active Flavour Substances Thujone and Camphor in Foods and Medicines Containing Sage (*Salvia officinalis* L.). Chem. Cent. J..

[44] Pelkonen O., Abass K., Wiesner J. (2013). Thujone and Thujone-Containing Herbal Medicinal and Botanical Products: Toxicological Assessment. Regul. Toxicol. Pharmacol..

[45] Hailfinger S., Jaworski M., Marx-Stoelting P., Wanke I., Schwarz M. (2007). Regulation of P53 Stability in P53 Mutated Human and Mouse Hepatoma Cells. Int. J. Cancer.

[46] Massimi M., Cardarelli S., Galli F., Giardi M.F., Ragusa F., Panera N., Cinque B., Cifone M.G., Biagioni S., Giorgi M. (2017). Increase of Intracellular Cyclic AMP by PDE4 Inhibitors Affects HepG2 Cell Cycle Progression and Survival. J. Cell Biochem..

[47] AAT Bioquest Quest IC50 Calculator. https://www.aatbio.com/tools/ic50-calculator.

[48] Ara C., Conti Devirgiliis L., Massimi M. (2004). Influence of Retinoic Acid on Adhesion Complexes in Human Hepatoma Cells: A Clue to Its Antiproliferative Effects. Cell Commun. Adhes..

[49] Pollio A., Zarrelli A., Romanucci V., Di Mauro A., Barra F., Pinto G., Crescenzi E., Roscetto E., Palumbo G. (2016). Polyphenolic Profile and Targeted Bioactivity of Methanolic Extracts from Mediterranean Ethnomedicinal Plants on Human Cancer Cell Lines. Molecules.

[50] Kim E.M., Jung C.H., Kim J., Hwang S.G., Park J.K., Um H.D. (2017). The P53/P21 Complex Regulates Cancer Cell Invasion and Apoptosis by Targeting Bcl-2 Family Proteins. Cancer Res..

[51] Mantovani F., Collavin L., Del Sal G. (2019). Mutant P53 as a Guardian of the Cancer Cell. Cell Death Differ..

[52] Mashimo M., Onishi M., Uno A., Tanimichi A., Nobeyama A., Mori M., Yamada S., Negi S., Bu X., Kato J. (2021). The 89-KDa PARP1 Cleavage Fragment Serves as a Cytoplasmic PAR Carrier to Induce AIF-Mediated Apoptosis. J. Biol. Chem..

[53] Jung E.J., Lee W.S., Paramanantham A., Kim H.J., Shin S.C., Kim G.S., Jung J.M., Ryu C.H., Hong S.C., Chung K.H. (2020). P53 Enhances *Artemisia annua* L. Polyphenols-Induced Cell Death through Upregulation of P53-Dependent Targets and Cleavage of Parp1 and Lamin a/c in HCT116 Colorectal Cancer Cells. Int. J. Mol. Sci..

[54] Ahmed E.A., Abu Zahra H., Ben Ammar R., Mohamed M.E., Ibrahim H.I.M. (2022). Beta-Caryophyllene Enhances the Anti-Tumor Activity of Cisplatin in Lung Cancer Cell Lines through Regulating Cell Cycle and Apoptosis Signaling Molecules. Molecules.

[55] Chirumbolo S., Bjørklund G. (2017). The Sesquiterpene α-Bisabolol in the Adipocyte−cancer Desmoplastic Crosstalk: Does It Have an Action on Epithelial−mesenchymal Transition Mechanisms?. Int. J. Clin. Oncol..

[56] Arafat K., Al-Azawi A.M., Sulaiman S., Attoub S. (2023). Exploring the Anticancer Potential of Origanum Majorana Essential Oil Monoterpenes Alone and in Combination against Non-Small Cell Lung Cancer. Nutrients.

[57] Balahbib A., El Omari N., Hachlafi N.E., Lakhdar F., El Menyiy N., Salhi N., Mrabti H.N., Bakrim S., Zengin G., Bouyahya A. (2021). Health Beneficial and Pharmacological Properties of P-Cymene. Food Chem. Toxicol..

[58] Lenis-Rojas O.A., Robalo M.P., Tomaz A.I., Carvalho A., Fernandes A.R., Marques F., Folgueira M., Yánez J., Vázquez-García D., López Torres M. (2018). RuII(p-Cymene) Compounds as Effective and Selective Anticancer Candidates with No Toxicity in Vivo. Inorg. Chem..

[59] Pudełek M., Catapano J., Kochanowski P., Mrowiec K., Janik-Olchawa N., Czyż J., Ryszawy D. (2019). Therapeutic Potential of Monoterpene α-Thujone, the Main Compound of *Thuja occidentalis* L. Essential Oil, against Malignant Glioblastoma Multiforme Cells in Vitro. Fitoterapia.

[60] Yang Y., Zhang Z., Li S., Ye X., Li X., He K. (2014). Synergy Effects of Herb Extracts: Pharmacokinetics and Pharmacodynamic Basis. Fitoterapia.

[61] Caesar L.K., Cech N.B. (2019). Synergy and Antagonism in Natural Product Extracts: When 1 + 1 Does Not Equal 2. Nat. Prod. Rep..

