# Supplementary figures and images for: Potential Antiproliferative and Antimetastatic Effects of Artemisia eriantha: An In Vitro Study Focused on Hepatocarcinoma Cells
Source: Biology (Basel). 2024 Nov 28;13(12):985. doi: 10.3390/biology13120985 (PMC11727042; doi:10.3390/biology13120985)

# Original WB (HepG2)

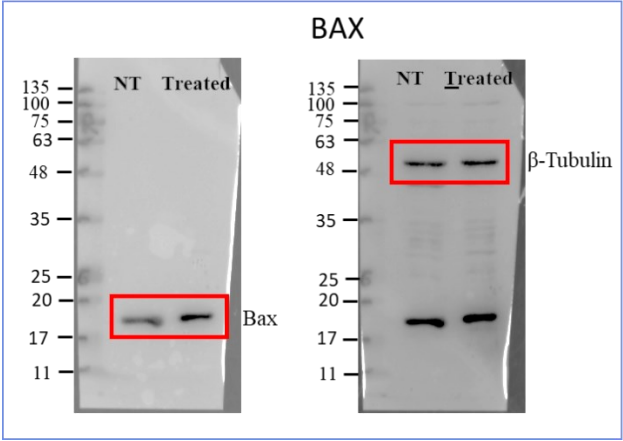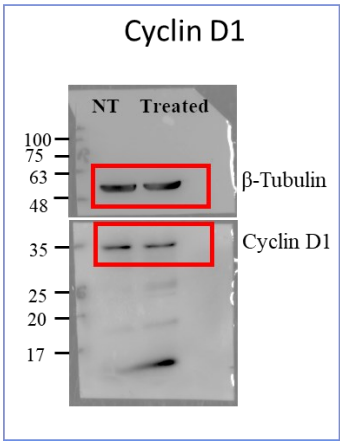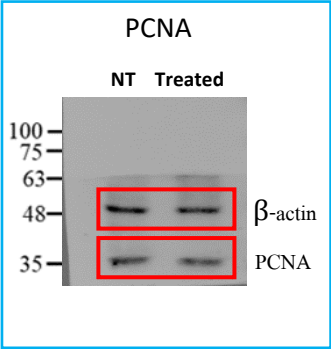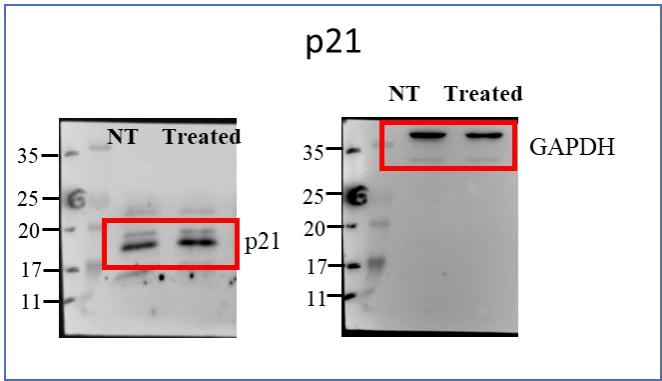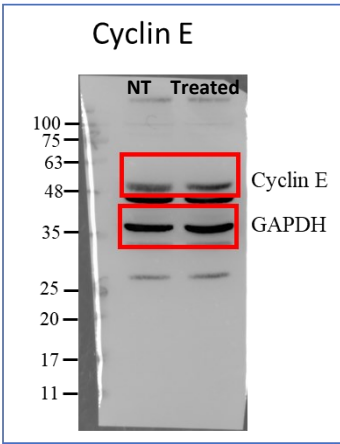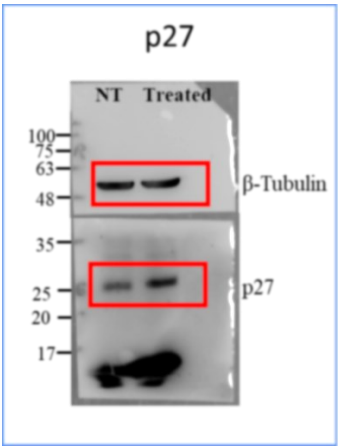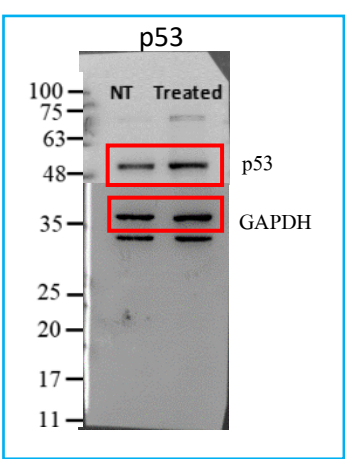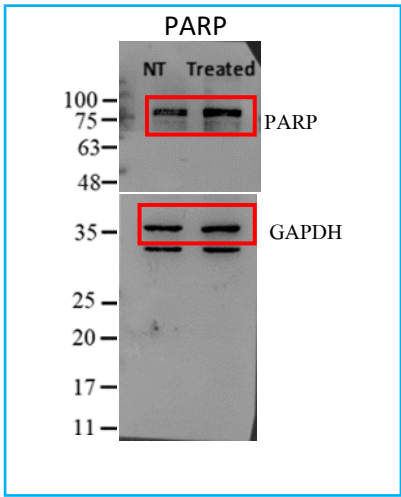

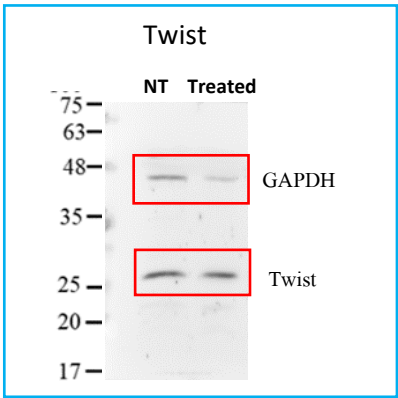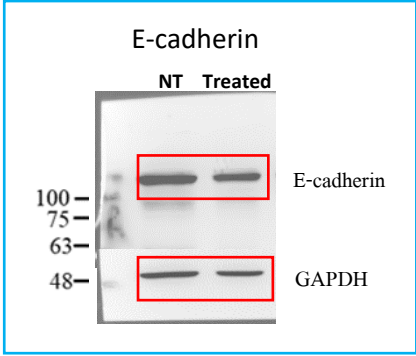

Original WB (Huh7)

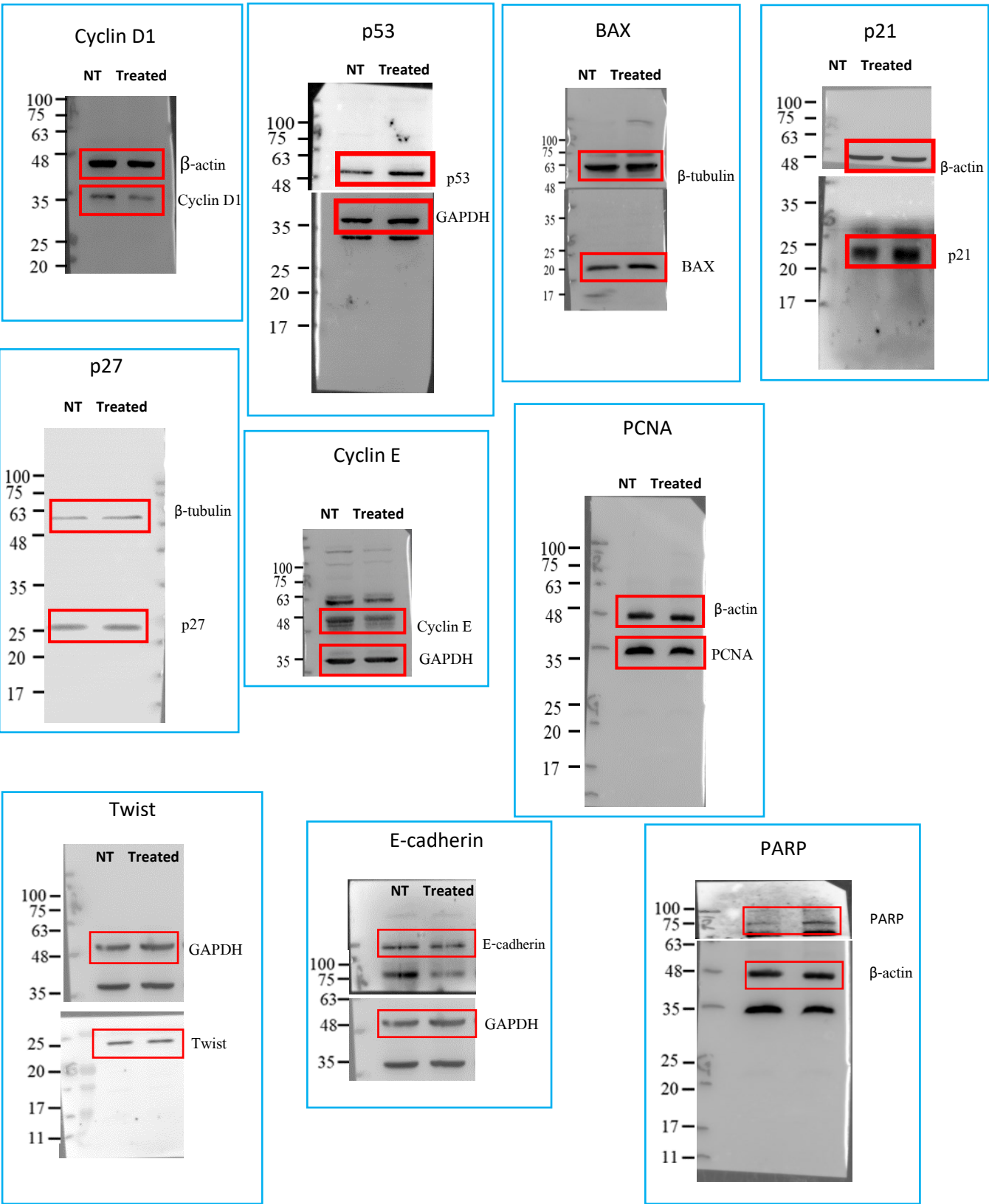

Supplement: Supplementary file 1 [file biology-13-00985-s001.zip › biology-3249101-supplementary.pdf]
